# Dry season refugia for anopheline larvae and mapping of the seasonal distribution in mosquito larval habitats in Kandi, northeastern Benin

**DOI:** 10.1186/1756-3305-7-137

**Published:** 2014-03-31

**Authors:** Renaud Govoetchan, Virgile Gnanguenon, Euloge Ogouwalé, Frédéric Oké-Agbo, Roseric Azondékon, Arthur Sovi, Roseline Attolou, Kefilath Badirou, Ramziyath Agbanrin Youssouf, Razaki Ossè, Martin Akogbéto

**Affiliations:** 1 Centre de Recherche Entomologique de Cotonou (CREC), 06 BP 2604 Cotonou, Bénin; 2 Faculté des Sciences et Techniques, Université d’Abomey Calavi Calavi, Bénin; 3 Département de Géographie, Université d’Abomey Calavi Calavi, Bénin; 4 University of Massachusetts Amherst, Amherst, USA

**Keywords:** Larvae, Habitats, Anopheline, Drought, Refugia, Breeding

## Abstract

**Background:**

The dynamics of mosquito populations depends on availability of suitable surface water for oviposition. It is well known that suitable management of mosquito larval habitats in the sub-Saharan countries, particularly during droughts, could help to suppress vector densities and malaria transmission. We conducted a field survey to investigate the spatial and seasonal distribution of mosquito larval habitats and identify drought-refugia for anopheline larvae.

**Methods:**

A GIS approach was used to identify, geo-reference and follow up longitudinally from May 2012 to May 2013, all mosquito breeding sites in two rural sites (Yondarou and Thui), one urban (Kossarou), and one peri-urban (Pèdè) site at Kandi, a municipality in northeastern Benin. In Kandi, droughts are excessive with no rain for nearly six months and a lot of sunshine. A comprehensive record of mosquito larval habitats was conducted periodically in all sites for the identification of drought-refugia of anopheline larval stages. With geospatialisation data, seasonal larval distribution maps were generated for each study site with the software ArcGIS version 10.2.

**Results:**

Overall, 187 mosquito breeding sites were identified of which 29.95% were recorded during drought. In rural, peri-urban and urban sites, most of the drought-refugia of anopheline larvae were domestic in nature (61.54%). Moreover, in rural settings, anopheline larvae were also sampled in cisterns and wells (25% of larval habitats sampled during drought in Yondarou and 20% in Thui). The mapping showed a significant decrease in the spatial distribution of mosquito larval habitats in rural, peri-urban and urban sites during drought, except in Yondarou (rural) where the aridity did not seem to influence the distribution of larval habitats.

**Conclusion:**

Our data showed that the main drought-refugia of anopheline larvae were of a domestic nature as well as wells and cisterns. A suitable management of mosquito larvae in sub-Saharan countries, particularly during droughts, should target such larval habitats for a meaningful impact on the dynamics of mosquito populations and malaria transmission.

## Background

Malaria is a major cause of global morbidity and mortality, with most of the burden being in sub-Saharan Africa [1,2]. More than 90% of recorded malarial deaths occur in Africa among the most vulnerable low immune response individuals, such as children under five years old and pregnant women [3,4]. And despite many efforts from National Malaria Control Programs (NMCP) and support from major donors to eradicate the disease through various vector control interventions, malaria continues to be a major/important public health problem.

In Benin, malaria is the leading reason for consultation and hospitalization in healthcare centers. In Kandi, a municipality of northeastern Benin, the malaria incidence in 2012 was 28.55% well above the national average of 14.6% [5]. In this location, the dry season is very severe and lasts about six months yet several malaria cases are diagnosed during this drought period. The healthcare registries in the Yondarou health center (rural Kandi) indicate that between 2008 and 2012, clinical malaria cases were estimated at 15 to 30% of all consultation motives during drought periods of which 56.64% were children under 10 years (Govoetchan, personal communication). Although these unexpected cases could be linked to relapse or cases of imported malaria, the possibility of recent infection is not ruled out in the light of the magnitude of the prevalence and the sedentary status of local people and especially children. It is therefore important to investigate the factors contributing to this observed pattern.

Several studies have shown that malaria infection is influenced by environmental factors such as temperature, rainfall, humidity and elevation. In tropical settings, temperature and rainfall conditions are nearly always favourable for the development of *Anopheles* mosquitoes, which are the intermediate hosts in the transmission of malaria parasites [6,7]. According to Martin and Lefebvre (1995), rain is generally synonymous with new mosquito breeding sites. However, rain can also destroy the existing breeding sites: heavy rains can transform basins to streams, hinder the development of eggs and larvae, or simply eject them from water. Conversely, extreme drought conditions lead to the evaporation of most of the conventional mosquito breeding sites resulting in lower mosquitoes’ abundance [8-12].

A potentially important target for malaria vector control is anopheline larvae. Source reduction through modification of larval habitats was the key to malaria eradication efforts in the United States, Israel, and Italy [13,14]. It is conceivable that suitable management of larval habitats in the sub-Saharan countries, particularly during the dry seasons, could help to suppress vector densities and malaria transmission. In order to undertake effective strategies against *Anopheles* exposure and malaria transmission, an important primary task is to obtain an in-depth knowledge of the spatial and temporal distribution of breeding sites. Unfortunately, there are challenges involved with larval sampling from aquatic habitats in the field, particularly when many larval habitats are not permanent [15].

Nowadays, Geographical Information Systems (GIS) have become essential tools for analysis of the temporal and spatial distribution of disease and vectors [13,16,17]. In this study, we applied the GIS approach to identify, geo-reference and follow up longitudinally all mosquito species breeding sites in rural and urban Kandi. In addition, we mapped the spatial and seasonal distribution of breeding sites and we examined the diversity in mosquito larval habitats, especially in anophelines throughout the year in order to access the situation during the dry season, a period supposed to be of very low or zero malaria transmission.

## Methods

### Site description

This study was carried out in Kandi (11 ° 07 ′43 ″N, 2 ° 56′ 13 E), a northeastern municipality of Benin. Kandi is under a Sudanian climate with a dry season from November to April and a wet season from May to October. In the dry season, temperatures are very high and can reach up to 45°C. The drought is severe with no rain for nearly six months and much sunshine. Our data collection was conducted in two rural areas (Yondarou and Thui), one peri-urban area (Pèdè) and one urban area (Kossarou). Overall, the sites are of relatively small surface area (under 1 km^2^). The option of site selection based on small surface area was done with the core aim to ensure that the whole surface can easily be crisscrossed and prospected. Of all the 4 sites, Kossarou had the largest surface area (652.423 m^2^) while Yondarou had the smallest (187.278 m^2^). In Thui and Pèdè, the surface areas are estimated at 620.164 m^2^ and 400.928 m^2^ respectively.

### Identification and geo-positioning of mosquito breeding sites

The data collection was carried out for one year (from May 2012 to April 2013) in Yondarou, Pèdè, Kossarou and Thui. Samplings were carried out exhaustively every two months during the wet season (May 2012 to October 2012), and each month during the dry season (November 2012 to April 2013). Monthly investigations were conducted during the dry season to ensure the identification and listing of all aquatic habitats that could potentially support mosquito breeding during periods of severe aridity. At each study site, all aquatic habitats were explored using a dipper (60 cm^3^ of volume) and any habitat harboring at least one mosquito larva was identified as a positive breeding site and its nature was recorded and geo-referenced. After a record of breeding site, a taxonomic identification of larvae found was performed to the genus level [18].

### Collection method

Data was collected using tablets Samsung Galaxy Tab® 10.1. An electronic survey form was created for this purpose with the ODK (Open Data Kit) collect software, which enabled the automatic recording of data in the field. The survey form allows for instant recording of both GPS (Global Positioning System) coordinates and entomological data for positive larval habitats identified. At the end of each study visit, the data was transferred directly to a cloud server in order to ensure their backup and traceability.

### Mapping of the seasonal distribution of mosquitoes

All GPS coordinates of identified mosquito breeding sites were projected onto a map. In each location, we developed a map of the distribution of mosquito larval habitats for the wet season, and another for the dry season. Mapping mosquito breeding sites aimed at seeking an overview of the geospatialisation of breeding sites, especially that of anophelines. Through this mapping, we investigated the geospatial distribution of the anopheline residual larval habitats that enabled the maintenance of malaria transmission during arid periods (dry season) supposed to be of scarce mosquito bites. In order to achieve a suitable and effective control of malaria vectors, such mosquito larval habitats should be targeted. The mapping was performed with the software ArcGIS version 10.2.

### Data analysis

Percentages for the various larval habitats, the genera of mosquitoes, and the anopheline larval habitats specific to the dry season were pairwise-compared using the multiple comparison test of proportions [19], with the method of adjustment of p-value of Holm [20]. Confidence intervals were calculated using the exact binomial method (small sample size) and the normal approximation to the binomial distribution method (large sample size) for the calculation of proportions’ confidence intervals [21]. Data analyses were performed using the software R-2.15.2 [22].

## Results

### Dry season refugia for mosquito larvae

A total of 187 mosquito larval habitats were identified in Yondarou, Pèdè, Kossarou and Thui between May 2012 and April 2013 of which 29.95% were recorded during the dry season (Table 1). In all the study sites, the diverse breeding sites recorded depended on the seasons. In the wet season (May-October 2012), rainwater collections and various holes were the majority constituting together 51.43% of all larval habitats recorded in Yondarou, 88.57% in Pèdè, 76.92% in Kossarou and 100% in Thui. However, during the long drought that lasts about six months (November 2012-April 2013), these classical mosquito breeding sites disappeared altogether. The drought-refugia for mosquito breeding were mainly the household canaries, jars, flower pots that we defined as domestic larval habitats. Moreover, we identified cisterns for water supplies and wells as typical refugia of mosquitoes breeding in drought.

**Table 1:** Seasonal diversity in larval habitats of mosquitoes in rural (Yondarou, Thui), peri-urban (Pèdè) and urban (Kossarou) Kandi

**Study sites**	**Nature of larval habitats**	**Wetseason**			**Dry season**			**Total**		
		**Total 1**	**%**	**CI-95%**	**Total 2**	**%**	**CI-95%**	**Total**	**%**	**CI-95%**
**Yondarou (rural)**	Rain water collections	13	37.14^a.b^	[21.47- 55.08]	0	0.00^a^	[00.00- 11.95]	13	20.31^a^	[11.28- 32.22]
	Various holes	5	14.29^b.c^	[04.81- 30.26]	0	0.00^a^	[00.00- 11.95]	5	7.81^b^	[02.53- 17.30]
	In shallow aquifer	1	2.86^c^	[00.07- 14.92]	0	0.00^a^	[00.00- 11.95]	1	1.56^b^	[00.04- 08.40]
	Domestic habitats	15	42.86^b^	[26.32- 60.65]	21	72.41^b^	[34.85- 98.73]	36	56.25^a.c^	[09.90- 81.59]
	Cisterns/Wells	0	0.00^c^	[00.00- 10.00]	2	6.90^a^	[00.85- 22.77]	2	3.13^b^	[00.38- 10.84]
	Around public standpipes	0	0.00^c^	[00.00- 10.00]	2	6.90^a^	[00.85- 22.77]	2	3.13^b^	[00.38- 10.84]
	Polluted water collections	0	0.00^c^	[00.00- 10.00]	1	3.45^a^	[00.09- 17.77]	1	1.56^b^	[00.05- 08.40]
	Others	1	2.86^c^	[00.07- 14.92]	3	10.34^a^	[02.19- 27.35]	4	6.25^b^	[01.73- 15.24]
	Total	35	100	-	29	100.00	-	64	100	-
**Thui (rural)**	Rain water collections	7	77.78^a^	[39.99- 97.19]	0	0.00^a^	[00.00- 40.96]	7	43.75^a^	[19.75- 70.12]
	Various holes	2	22.22^a.b^	[02.81- 60.01]	0	0.00^a^	[00.00- 40.96]	2	12.50^b^	[01.67- 99.30]
	Domestic habitats	0	0.00^b^	[00.00- 33.63]	3	42.86^a.b^	[09.90- 81.59]	3	18.75^a.b^	[04.05- 45.65]
	Cisterns/Wells	0	0.00^b^	[00.00- 33.63]	3	42.86^a.b^	[09.90- 81.59]	3	18.75^a.b^	[04.05- 45.65]
	Polluted water collections	0	0.00^b^	[00.00- 33.63]	1	14.29^a.c^	[00.36- 57.87]	1	6.25^b.c^	[00.19- 30.23]
	Total	9	100	-	7	100	-	16	100	-
**Pèdè (peri-urban)**	Rain water collections	29	82.86^a^	[66.35- 93.44]	0	0.00^a^	[00.00- 30.85]	29	64.44^a^	[48.78- 78.13]
	Various holes	2	5.71^b^	[00.70- 19.16]	0	0.00^a^	[00.00- 30.85]	2	4.44^b^	[00.54- 15.15]
	In shallow aquifer	1	2.86^b^	[00.70- 14.92]	0	0.00^a^	[00.00- 30.85]	1	2.22^b^	[00.06- 11.77]
	Domestic habitats	0	0.00^b^	[00.00- 10.00]	0	0.00^a^	[00.00- 30.85]	0	0.00^c^	[00.00- 07.87]
	Cisterns/Wells	0	0.00^b^	[00.00- 10.00]	7	70.00^b^	[34.75- 93.33]	7	15.56^d^	[06.49- 29.46]
	Around public standpipes	2	5.71^b^	[00.70- 19.16]	3	30.00^a.b^	[06.67- 65.25]	5	11.11^d^	[03.71- 24.05]
	Polluted water collections	1	2.86^b^	[00.70- 14.92]	0	0.00^a^	[00.00- 30.85]	1	2.22^b^	[00.06- 11.77]
	Total	35	100	-	10	100	-	45	100	-
**Kossarou (urban)**	Rain water collections	34	65.38^a^	[50.91- 78.03]	0	0.00^a^	[00.00- 30.85]	34	54.84^a^	[41.68- 67.52]
	Various holes	6	11.54^b^	[04.35- 23.44]	0	0.00^a^	[00.00- 30.85]	6	9.68^b^	[03.63- 19.88]
	Domestic habitats	11	21.15^b.c^	[11.70- 46.42]	0	0.00^a^	[00.00- 30.85]	11	17.74^b.c^	[08.42- 31.01]
	Cisterns/Wells	0	0.00^d^	[00.00- 06.85]	5	50.00^b^	[18.71- 81.29]	5	8.06^b^	[02.67- 17.83]
	Polluted water collections	1	1.92^d^	[00.05- 16.26]	5	50.00^b^	[18.71- 81.29]	6	9.68^b^	[03.63- 19.88]
	Total	52	100	-	10	100	-	62	100	-
	**Total**	**131**	**70.05**	[63.49- 76.62]	**56**	**29.95**	[23.38- 36.51]	**187**	100	

*Types of larval breeding habitats that were not mentioned at a study sites (Table 1) should be considered as not present in this location.

CI-95%: 95%-Confidence Interval.

^a,b,c,d^Values with the same superscript do not differ significantly at α = 0.05.

The type of habitat recorded during the drought period depended on the level of urbanization. During the dry season, the majority of larval habitats sampled in rural sites (Yondarou and Thui) were of domestic nature, whereas in urban (Kossarou) and peri-urban (Pèdè) sites, most of the drought-refugia for mosquito breeding was cisterns and wells. Domestic mosquito larval habitats represented, 72.41% and 42.86% respectively, of all breeding sites recorded in Yondarou and Thui. Conversely, cisterns and wells constituted, 50% and 70% respectively of larval habitats recorded during the dry season in Kossarou and Pèdè. However, larval breeding sites were also sampled during drought periods around public standpipes in Pèdè (30%) and Yondarou (6.90%) and in some collections of polluted water in Yondarou (3.45%), Kossarou (50%) and Thui (14.29%).

### Identification of mosquito larvae from the different habitat types

The main genera of mosquito we found in the larval habitats were *Anopheles*, *Culex* and *Aedes* (Table 2). The breeding sites were either exclusive (only a record of a single mosquito genus), or mixed (having two or three genera of mosquito found in sympatry). The prevailing mosquito genus in the larval habitats depended on the seasons of the year and the nature of study site (rural, peri-urban or urban).

**Table 2:** Genera of mosquitoes found in thelarval habitats in rural (Yondarou, Thui), peri-urban (Pèdè) and urban (Kossarou) Kandi

**Sites**	**Genus of mosquitoes’ larvae**		**Wetseason**			**Dry season**		**Total**		
		**Total 1**	**%**	**CI-95%**^**4**^	**total 2**	**%**	**CI-95%**	**N**	**%**	**CI-95%**
**Yondarou (rural)**	*An.*^1^	17	48.57^a^	[31.38-66.01]	2	6.90^a.b^	[00.85-22.77]	19	29.69^a^	[18.91-42.42]
	*Cx.*^2^	6	17.14^b.c^	[06.56-33.65]	8	27.58^a.c^	[12.73-47.24]	14	21.88^a^	[12.51-33.97]
	*Ae.*^3^	0	0.00^c^	[00.00-10.00]	1	3.45^b^	[00.09-17.76]	1	1.56^b^	[00.04-08.40]
	*An.* and *Cx.*	10	28.57^a.b^	[14.64-46.30]	15	51.72^c^	[32.53-70.55]	25	39.06^a^	[27.10-52.07]
	*An.* and *Ae.*	1	2.86^c^	[00.07-14.92]	2	6.90^a.b^	[00.85-22.77]	3	4.69^b^	[00.98-13.09]
	*Cx.* and *Ae.*	1	2.86^c^	[00.07-14.92]	1	3.45^b^	[00.09-17.76]	2	3.12^b^	[00.38-10.84]
	Total	35	100.00	[90.00-100.0]	29	100.00	[90.00-100.0]	64	100.00	[90.00-100.0]
**Thui (rural)**	*An.*	3	33.33^a^	[07.49-70.07]	1	14.29^a^	[00.36-57.87]	4	25.00^a.b^	[07.27-52.38]
	*Cx.*	0	0.00^a^	[00.00-33.63]	2	28.57^a^	[03.67-70.96]	2	12.50^a.b^	[01.55-38.35]
	*Ae.*	6	66.67^a^	[29.93-92.51]	4	57.14^a^	[18.41-90.10]	10	62.50^b^	[35.43-84.80]
	Total	9	100.00	[90.00-100.0]	7	100.00	[90.00-100.0]	16	100.00	[90.00-100.0]
**Pèdè(peri-urban)**	*An.*	28	80.00^a^	[51.75-97.73]	3	30.00^a.b^	[06.67-65.25]	31	68.89^a^	[38.13-86.93]
	*Cx.*	1	2.86^b^	[00.07-14.92]	0	0.00^a^	[00.00-30.85]	1	2.22^b^	[00.03-14.65]
	*An.* and *Cx.*	5	14.28^b^	[08.86-37.77]	7	70.00^b^	[34.75-93.33]	12	26.67 ^c^	[12.02-47.06]
	*An.* and *Ae.*	1	2.86^b^	[00.07-14.92]	0	0.00^a^	[00.00-30.85]	1	2.22^b^	[00.03-14.65]
	Total	35	100.00	[90.00-100.0]	10	100.00	[90.00-100.0]	45	100.00	[90.00-100.0]
**Kossarou (urban)**	*An.*	19	36.54^a^	[23.62-51.04]	2	20.00^a^	[00.52-55.61]	21	33.87^a^	[22.33-47.01]
	*Cx.*	24	46.15^a^	[32.23-60.53]	7	70.00^a.b^	[34.75-93.33]	31	50.00^a^	[37.02-62.98]
	*Ae.*	1	1.92^b^	[00.05-10.26]	0	0.00^a^	[00.00-30.85]	1	1.61^b^	[00.04-08.66]
	*An.* and *Cx.*	8	15.39^c^	[06.88-28.08]	1	10.00^a^	[00.25-44.50]	9	14.52^c^	[06.86-25.78]
	Total	52	100.00	[90.00-100.0]	10	100.00	[90.00-100.0]	62	100.00	[90.00-100.0]
	Total	131	**70.05**	[63.49-76.62]	**56**	**29.95**	[23.38-36.51]	**187**	100.00	[90.00-100.0]

*data with different letters are statistically different at α = 0.05.

^1^*Anopheles.*

^2^*Culex.*

^3^*Aedes.*

^4^ 95%-Confidence Interval.

In rural (Yondarou and Thui) and peri-urban (Pèdè) sites, *Anopheles*-exclusive larval habitats were the most sampled during the wet season (60.79%). During this season, very few larval habitats of *Culex* mosquitoes were recorded in these areas (17.14% in Yondarou, 0% in Thui and 2.86% in Pèdè). Conversely in urban locations (Kossarou), *Culex* mosquito breeding sites were most abundant during the wet season (46.15%) followed by *Anopheles* (36.54%).

With regard to the dry season, we observed that in rural, peri-urban and urban sites, there was a significant decrease in *Anopheles*-exclusive larval habitats and an increase in larval habitats of *Culex*. During such drought periods, *Anopheles* larvae were mainly sampled in mixed breeding sites of mosquitoes (multi-genera breeding sites) especially in sympatry with *Culex* (51.72% and 70%, respectively, in Yondarouand in Pèdè).

### Refugia of anopheline larvae in drought periods

A total of 26 anopheline larval habitats (exclusive and mixed) were identified in the 4 study sites during the dry season (November 2012-April 2013). Data analysis showed that in rural, peri-urban and urban sites most of drought-refugia of anopheline larvae were of domestic nature (61.54%) (Table 3). Moreover, in rural settings, anopheline larvae were also sampled in cisterns and wells (25% of larval habitats sampled during drought in Yondarou and 20% in Thui). Polluted water collections and perimeters around standpipes were also identified as important drought-refugia sites for anopheline breeding.

**Table 3:** Habitats in anopheline larvae during drought periods in rural (Yondarou, Thui), peri-urban (Pèdè) and urban (Kossarou) Kandi

**Village**		**Variousholes**	**Domestic habitat**	**Cistern/Well**	**Polluted water collection**	**Around public standpipes**	**Total**
**Yondarou**	N	1	6	3	0	2	12
	%	8.30%	50.00%	25.00%	0.00%	16.70%	100.00%
**Thui**	N	0	3	1	1	0	5
	%	0.00%	60.00%	20.00%	20.00%	0.00%	100.00%
**Pede**	N	0	5	0	0	1	6
	%	0.00%	83.30%	0.00%	0.00%	16.70%	100.00%
**Kossarou**	N	0	2	0	1	0	3
	%	0.00%	66.70%	0.00%	33.30%	0.00%	100.00%
**Total**	**N**	**1**	**16**	**4**	**2**	**3**	**26**
	**%**	**3.85%**^**a**^	**61.54%**^**b**^	**15.38%**^**c**^	**7.69%**^**a, d**^	**11.54%**^**c**^	**100.00%**

*data with different letters are statistically different.

### Mapping of the seasonal distribution in mosquito larval habitats

**Figure 1:**
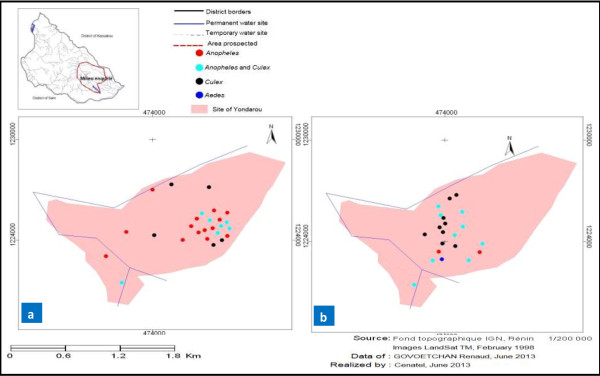
Geospatial occurrence of mosquito larval habitats in Yondarou during (a) the wet and (b) dry seasons.

**Figure 2:**
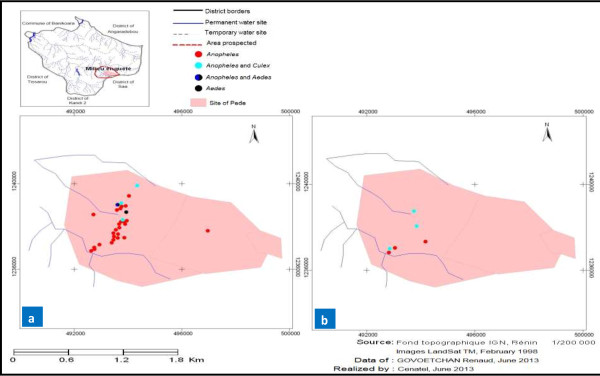
Geospatial occurrence of mosquito larval habitats in Pèdè during (a) the wet and (b) dry seasons.

**Figure 3:**
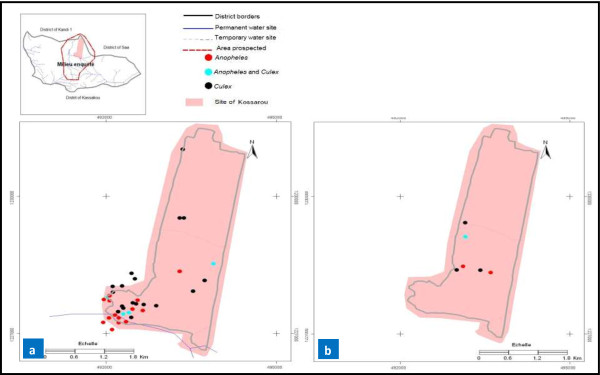
Geospatial occurrence of mosquito larval habitats in Kossarou during (a) the wet and (b) dry seasons.

**Figure 4:**
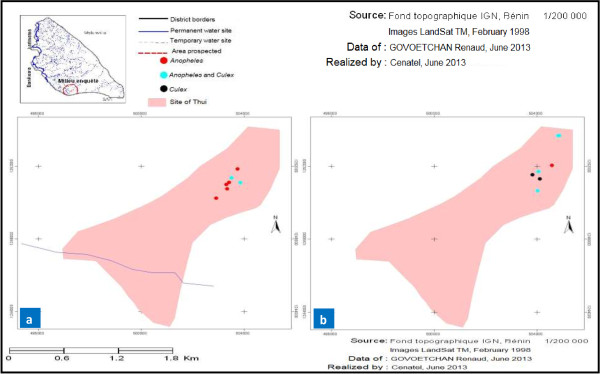
Geospatial occurrence of mosquito larval habitats in Thui during (a) the wet and (b) dry seasons.

A record of GPS coordinates of larval habitats allowed us to develop the seasonal dispersion maps for each study site. Overall, mapping showed a proliferation of mosquito larval habitats during the wet season in both rural (Yondarou and Thui) and peri-urban (Pèdè) locations (Figures 1a, 2a, 3a and 4a). But the situation was different during drought, with very few mosquito breeding sites mapped for rural sites (Figure 1b and 4b) and peri-urban sites (Figure 2b), except in Yondarou where the spatial analysis showed that the drought did not seem to fundamentally influence the distribution of larval habitats that we observed in the wet season (Figure 1a and 1b). In this village, we noted very little difference between the number of larval habitats in dry and wet seasons but the number of *Anopheles* larval habitats (exclusive or mixed) appeared to have significantly decreased in dry season.

## Discussion

An integrated management of larval habitats in sub-Saharan countries, particularly during the dry seasons, could help suppress vector densities and malaria transmission. A study to target the distribution of mosquito breeding sites, however, involves careful research of larval stages of mosquitoes over the entire surface of the identified sites, including in vegetation.

Our data showed that the proliferation of mosquito larval stages during the dry season is lower than in the wet season. Many studies have reported a high anopheline density just after the first rains. The explanation proposed by Simard *et al.* (2000) and Yaro *et al.* (2012) indicates that anophelines that survive during the dry seasons undertake a physiological reorganization through a declining reproductive performance and keep eggs in their ovaries because of lack of larval habitats for laying [23,24]. Then, with the first rains, more larval habitats proliferate and aestivating mosquitoes can lay their eggs. The availability of larval habitats is a likely prerequisite for oviposition in gravid females aestivating. In addition, in wet periods, the regularity of rainfall associated with lower sunshine extends the existence of breeding sites. That would be the reason why from May to October 2012, breeding sites were regularly identified in rural, peri-urban and urban settings. But upon the return of the drought, the number of larval habitats drastically decreased in all study sites (rural, peri-urban and urban locations) and the nature of larval habitats depended fundamentally on the type of location (rural *versus* peri-urban or urban locations). The larvae sampled during the dry season in rural settings were collected mainly from a domestic environment. This is probably due to the fact that in these locations, water supply is a major concern during droughts. Households do not have pipe borne water and most wells dry up between November and April. Thus, as the public standpipes are not always near their homes, the locals store up water reserves in jars and other containers. But unfortunately, containers and jars are not covered and then become potential oviposition sites for gravid and aestivating mosquitoes, and particularly anophelines in such rural locations. Regarding peri-urban and urban locations, the situation was not the same because pipe borne water is available in households so that water storage is not frequent. In such conditions, proper conservation of water reserves is important in order to decrease the frequency of domestic larval habitat in rural locations and on anopheline populations and malaria transmission during this period.

Three genera were sampled (*Anopheles*, *Aedes* and *Culex*). The hypothesis of the presence of other mosquito genera cannot be excluded. It is possible that other genera of mosquitoes exist in our study sites and were not collected using the dipper sampling method. In the wet season, *Anopheles* larvae were mostly sampled in rural and peri-urban locations, whereas *Culex* larvae were the majority in urban settings. Our data suggest that urbanization greatly influences the mosquito fauna. It is known that anophelines prefer transient breeding sites, which are usually not many in urban areas because of improved drainage. This is certainly the reason why, the risk of *Anopheles* biting and malaria transmission are lower in urban areas compared to rural areas as reported by Gardiner *et al.*(1984), Trape (1987), Watts *et al.*(1990), Fontenille *et al.*(1997), Gila *et al.*(2003), and Way *et al.*(2005), [25-30]. Regarding the dry season, the situation changed very little in urban settings and *Culex* mosquitoes were predominant, whereas in rural and peri-urban settings, anopheline larvae were mainly sampled in sympatric breeding sites. This could be due to the fact that, classical breeding sites of anophelines dried with the very high aridity so that gravid females choose to lay eggs in unusual habitats and already hosting larvae of other genera.

This confirms the relatively advanced urbanization of Kossarou, located in the city center of Kandi. The evolution of the density of *Anopheles* in exclusive and mixed larval habitats depended on the seasons. In all study sites, a higher density of *Anopheles* larvae were recorded during the rainy season, making the risk of malaria transmission real during this time of year.

In our mapping, we prospected for larval habitats up to 2 km beyond each study site’s boundaries. This is due to the fact that in *An. gambiae s.s.,* the ray of dispersion around the habitat of its emergence varies on average between 1 and 1.6 km [31]. Therefore, we assumed that mosquitoes emerging from a breeding site located outside of a village can, through dispersion, bite humans inside the village. This approach enabled us to include all the data from outside our target sites in order to properly identify the possible sources of mosquito breeding sites. However, it is important to note that the mapping of mosquito breeding sites depended on rainfall that prevailed at the time of data collection. This means that the graphical representations made especially during the wet season may change depending on rainfall data. To perform a complete mapping of mosquito larval habitats, it would be ideal to consider multi-year data to make sure that changes in rainfall patterns are taken into account. However, our mapping displayed an important pattern in the distribution of larval habitats in Kossarou, Pèdè, Yondarou and Thui that can be of great value in the application of malaria vector control.

Nevertheless, our study has limitations. The larval identification was not performed until species determination. This is a limitation since it is not clear whether the *Anopheles* larvae collected in the study sites were actually malaria vectors or not. However, the high prevalence of malaria cases recorded during droughts in the healthcare centers of Kandi (Govoetchan, personnal communication) suggests that *Anopheles* vectors would be predominant. Moreover, the data of Govoetchan *et al.* (unpublished data) on mosquito diversity in the municipality of Kandi showed that *An. gambiae*, the main vector of malaria in Africa, represents 99.40% of anopheline adults collected in Kossarou, Pèdè, Yondarou and Thui. Another potential limitation of this study is that we did not present the larval density in the drought-refugia of mosquitoes. However, since the main focus of this study was to seek atypical habitats, which maintain mosquito breeding when classical larval habitats have dried up, this limitation should not greatly affect interpretation of these results.

## Conclusion

Our data showed that mosquito larval habitats were abundant during the wet season but most of them disappeared, substituted by atypical larval habitats. Domestic jars and recipients, wells and cisterns were the main mosquito larval habitats identified during drought periods across rural, peri-urban and urban settings in the municipality of Kandi, northeastern Benin. These atypical habitats served to maintain mosquito breeding. A suitable management of mosquito larvae in sub-Saharan countries, particularly during droughts, targeting such larval habitats could have a meaningful impact on the dynamics of mosquito populations, and hence reduce the transmission burden of malaria.

## Competing interests

The authors declare that they have no competing interest.

## Authors’ contributions

RG and MA designed the study. RG, VG, AS, RA, KB and RAY carried out the field activities. RG, RA and FOA analyzed the data. RG drafted the manuscript. MA, EO, RA and RO critically revised the manuscript for intellectual content. All authors read and approved the final manuscript.
